# A Case of Symphyseotomy

**Published:** 1856-04

**Authors:** M. Maslieurat-Lagemard


### *A Case of Symphyseotomy.* By M. MASLIEURAT-LAGEMARD.

(Medico-Chir. Review, Oct. 1855; and Bull. Gen. de Ther. May, 1855.)

We copy Dr. Barne's abstract of this case.

CASE.—The subject of this case was a woman who had borne two children without anything remarkable occurring. In a third labor (November, 1847), the head was arrested, and symptoms of exhaustion set in; the forceps were tried several times in vain. Turning was then resorted to, the feet were brought down, but the head resisted all endeavors at extraction. The woman had now been three days in labor. Choice lay between Cæsarian section and symphyseotomy. The latter was selected. An incision was made in the median line of the symphsis, the cartilage was then divided by a blunt-pointed bistoury. The separation of the iliac bones was effected by pressing with each hand upon the antero-superior spines of the ilia. A slight crackling was heard in the sacroiliac articulations, and the two branches of the pubis were found sufficiently parted to admit the finger. As soon as this was done, a very gentle traction on the body of the fœtus sufficed to bring forth the head, and finish the labor. The child was dead. A bandage was applied round the pelvis, to bring the pubic bones together. Three days after, shivering and acute pains in the right leg set in; phlegmasia dolens became developed. The patient recovered, notwithstanding, and renewed her occupations at the end of fifteen days. M. Maslieurat Lagemard has seen her recently; she has been delivered again since of a living child, well-formed, without difficulty.

It is right to add that M. Maslieurat was unprovided with the instruments requisite for cephalotripsy or for excerebration.

[*Ranking's Abstract.*

